# Supplementary material for: Modelling approaches for predicting the distribution of skin NTDs: A systematic review
Source: PLoS Negl Trop Dis. 2026 May 4;20(5):e0013662. doi: 10.1371/journal.pntd.0013662 (PMC13155686; doi:10.1371/journal.pntd.0013662)
Supplement: S2 File — Extracted data for the 68 included studies. (DOCX) [file pntd.0013662.s002.docx]

| **No** | **Author, Year (ref)** | **Region** | **lead author’s institutional affiliation** | **Disease(s)** | **Model** | **Data source** | **True absence/ Pseudoabsence** | **Software** | **Environmental predictors** | **Selection of predictors** | **Performance and sensitivity analysis** | **Limitations** |
| --- | --- | --- | --- | --- | --- | --- | --- | --- | --- | --- | --- | --- |
| 1 | Rajabi M, el al,2016^1^ | Iran | Sweden | CL | G | passive surveillance | not stated | Repast (Java-based simulation), GIS | LULC, proximity to water, population density, and healthcare access. | Based on known ecological and socio-environmental factors​ | Morris and Sobol's methods | Uncertainties in rodent habitats, validation data limitations​ |
| 2 | Purse BV, et al,2017^2^ | South, Meso-America | UK | CL and VL | ENM | passive surveillance, literature | Pseudoabsence only | R statistical software | Climate data, land use datasets, and mammal richness datasets​ | Stepwise selection via cross-validation. | Cross-validation, AUC, Pearson correlation​ | Climate uncertainty, data biases, and unknown host-vector relationships​ |
| 3 | Karagiannis-Voules DA,2013^3^ | Brazil | Switzerland | CL and VL | G | Case notifications | not stated | R, WinBUGS | Climatic data and environmental data (LST, NDVI, EVI) | AIC: Gibbs selection in WinBUGS chose predictors for the Bayesian spatial model. | Sensitivity analysis; DIC, posterior checks, and cross-validation | limited by data quality, resolution mismatch, model complexity, and uncertainty |
| 4 | Wijerathna T,el al,2022^4^ | Rwanda | Sri Lanka | CL | ENM | Case notifications | Pseudoabsence only | R, QGIS | monthly temperature and rainfall data | Excluded variables based on the variance Inflation Factor (VIF). | AUC | Use background points instead of presence-absence data and recall bias by patients |
| 5 | King R, el al,2004^5^ | Colombia | USA | CL | G | Case notifications, passive survey | Pseudoabsence only | ArcView, TNTmips, MicroImages, Inc | Environmental and climatic temperature, LULC, elevation, and population. | Logistic regression model using land cover and elevation data, refined by ecological zones | Jackknife cross-validation, AUC, PPV sensitivity, specificity, Kappa. | Bias from underreporting, ecological variation, and validation challenges. |
| 6 | Ullah W,el al,2023^6^ | Pakistan | Pakistan | CL | G | passive surveillance | not stated | ArcGIS, R, SPSS | climate data and topography | Not stated | Not stated | lack of disease surveillance, data limitations |
| 7 | Zeb I,el al,2021^7^ | Pakistan | Pakistan | CL | G | passive surveillance | Pseudoabsence only | ArcGIS | Local environmental data (precipitation, temperature, LCLU, topography, vegetation) and census demographics. | Statistical analysis of CL incidence, spatial clustering, and environmental risk factors | Not stated | Data gaps, potential underreporting, and environmental variation |
| 8 | Shabanpour N,el al,2022^8^ | Iran | Iran | CL | G | passive surveillance | Pseudoabsence only | ArcGIS | Bioclimatic variables. | Statistical analysis of CL incidence and spatial clustering | Not stated | Data gaps, potential underreporting, and environmental variation |
| 9 | Jagadesh S,el al,2021^9^ | French Guiana​ | Switzerland | CL | ENM | Case notifications, passive surveillance | Pseudoabsence only | R, MaxEnt, QGIS​ | Data on temperature, precipitation, and humidity levels collected from local weather stations. | Based on statistical importance in the model​ | AUC and ROC curves​ | Not explicitly mentioned​ |
| 10 | Artun O,2019^10^ | Turkey | Turkey | CL | ENM | passive surveillance | Pseudoabsence only | MaxEnt, ArcMap | Bioclimatic and topographical variables | Selected based on correlation with CL distribution​ | Jackknife test, AUC, and ROC. | Bias in data collection, potential changes in environmental conditions​ |
| 11 | Adegboye OA,2017^11^ | Afghanistan | France | CL | ENM | Case notifications, passive surveillance | Pseudoabsence only | R, MaxEnt | Climatic variables | The Jackknife test for variable importance. | jackknife test, ROC, Log Score, AIC, AUC | Used only health facility data; varied landscapes and complex environments reduced accuracy. |
| 12 | Andreo V,2022^12^ | Argentina | Qatar | CL | ENM | Case notifications | Pseudoabsence only | GRASS, MaxEnt, QGIS,R program | Landsat 8 (2014–2016) data, map land cover, and detect changes using spectral indices and CVA. | Removed colinear variables, model refinement based on AUC, and tuned with a genetic algorithm. | Jackknife, AUC, sensitivity, specificity, cross-model validation. | presence-only data bias, moderate resolution, uncertain transmission sites, and few cases. |
| 13 | Pérez-Flórez M,el al,2016^13^ | Colombia | Argentina | CL | G | Case notifications | not stated | GeoBUGS, ArcMapWinBUGS, | Bioclimatic variables, elevation data, and population density | Checked collinearity, selected 31 predictors, and used stepwise analysis | Sensitivity with Gamma priors, DIC; assessed via posterior checks and trace plots. | Data gaps, possible measurement errors, and limited climate/land use variation modeling. |
| 14 | Seid A, el al,2014^14^ | Ethopia | Colombia | CL | ENM | research case | true absence | Stata/SE, ArcGIS | Bioclimatic variables, elevation and slope, land cover, and soil type data. | Predictors chosen from literature/expert input; variables with VIF > 10 excluded. | cross-validation, sensitivity, ROC-AUC, confusion matrix metrics, Kappa, threshold test | Data limits, linear model assumptions, and missing dynamic factors. |
| 15 | Chavy A,el al, 2019^15^ | French Guiana and Neotropical moist forests. | Ethiopia | CL | ENM | Case notifications | Pseudoabsence only | MaxEnt, ArcMap | Bioclimatic variables, environmental, anthropogenic, and spatial variables specific to French Guiana. | Used backward stepwise and jackknife tests; kept variables with >5% contribution. | AUC, omission rate, and comparison with a null model to evaluate reliability | Data gaps and complexity limited modeling. Human impact raised risk, but satellite data gave only rough insights. |
| 16 | B C,el al,2016^16^ | Tunisia | Tunisia | CL | ENM | Case notifications | Pseudoabsence only | ArcGIS, Maxent | Bioclimatic variables, topographic data (GTOPO30), and 300m land cover data (ESA). | Removed highly correlated (r ≥ 0.9) and low-contributing (<1%) predictors; favored extremes over means. | AUC, Cohen’s Kappa, and the Jackknife test. | lack of absence data, heavy reliance on environmental factors, and challenges in validating models on new data. |
| 17 | Artun O, 2019^17^ | Turkey | Turkey | CL | ENM | Case notifications | Pseudoabsence only | MaxEnt, ArcGIS | Altitude, slope, aspect, and bioclimatic variables on temperature and precipitation. | Checked multicollinearity with Pearson correlation; retained 11 uncorrelated variables. | AUC-ROC, jackknife, repeated cross-validation. | presence-only data, limited resolution, and assumed stable conditions, which may reduce accuracy over time. |
| 18 | Artun O,el al,2018^18^ | Turkey | Turkey | CL | ENM | Case notifications | Pseudoabsence only | MaxEnt, ARCMAP | Bioclimatic variables, including temperatures of driest and warmest periods. | Jackknife test is of variable importance. | Sensitivity, Jackknife test, AUC, and ROC curves. | The accuracy of the model heavily depended on the quality of the epidemiological and environmental data used. |
| 19 | ARTUN O,el al,2019^19^ | Turkey | Turkey | CL | ENM | Passive surveillance | Pseudoabsence only | MaxEnt, ArcGIS | Bioclimatic variables, 3 topographical variables (altitude, slope, aspect)​ | Retained four most important variables based on Jackknife test | Jackknife, ROC, AUCs | Bias in data collection, regional differences in climate and topography |
| 20 | Amro A,el al,2017^20^ | Libya | Palestine | CL | ENM | Passive surveillance | Pseudoabsence only | QGIS, R, MaxEnt​ | Bioclimatic variables , topographical, atmospheric. | Based on known ecological factors. | Jackknife test ,AUC-ROC. | Bias in data collection, regional climate variation​ |
| 21 | Meneguzzi VC,el al,2016^21^ | Brazil (Espírito Santo state)​ | Brazil | CL | ENM | Passive surveillance, survey, literature review | Pseudoabsence only | MaxEnt, ArcGIS ENMTools, ​ | Bioclimatic variables from WorldClim and elevation data from the Shuttle Radar Topography Mission (SRTM) | Based on correlation with CL distribution. | Schoener’s D for niche overlap, AUC, ROC. | Incomplete case reporting, Lack of true absence data, Temporal mismatch between climate ,case |
| 22 | Trájer AJ, el al, 2023^22^ | Western Asia | Hungary | CL | ENM | Case notifications, passive surveillance | not stated | not stated | climate projections, land cover, precipitation, and temperature as environmental variables. | Based on known ecological factors. | Not stated | Climate uncertainty, data limitations​ |
| 23 | Pigott D,el al, 2014^23^ | global | UK | CL | ENM | Case notifications, literature review | Pseudoabsence only | R | Climate data | Predictor importance based on how often each variable split trees in the BRT model. | AUC, sensitivity tested. | The study faced limitations due to pseudo-data use and spatial bias from underreporting. |
| 24 | Moo-Llanes DA,2016^24^ | Neotropical region | Mexico | CL | ENM | Literature review | Pseudoabsence only | MaxEnt, ENMtools | Bioclimatic variables | Excluded highly correlated , predictors, keeping nine for final model. | Partial-ROC, AUC. | biased data; no absence data or fine-scale details; climate and land use changes not fully included. |
| 25 | Ovalle-Bracho C, el al, 2019^25^ | Colombia | Colombia | CL, ML​ | G | Case notifications, survey, literature review | Pseudoabsence only | MaxEnt, ArcGIS, R​ | geographic data such as precipitation, temperature, elevation, vegetation, | Based on environmental relevance, statistical validation​, Add principal component analyses (PCA) | AUC and ROC | Data availability and underreporting of cases​ |
| 26 | Navi Z, el al,2024^26^ | Iran | Iran | CL & VL | G | Case notifications, literature review | Pseudoabsence only | ArcGIS, SPSS | Precipitation, temperature, and soil data from the Iranian Climatological Research Centre | Used ANOVA to identify statistically significant associations. | ANOVA | Limited availability of climatic data for some regions, reliance on molecular-based reports |
| 27 | Cadavid Restrepo AM,2023^27^ | American Samoa | Australia | LF^[[1]](#endnote-1)^ | G | Survey | true absence | R, Open BUGS, ArcGIS | LULC, Topographic, distance to water bodies, human population density | Stepwise selection using AIC and Bayesian Information Criterion | ROC, AUC | Spatial gaps in data collection, lack of detailed mosquito exposure data, limitations in diagnostic sensitivity |
| 28 | Cano J,el al, 2017^28^ | global | UK | LF | ENM | Survey, literature review | True Absence and Pseudoabsence | not stated | Climate, topography, vegetation, human population | Predictor importance | PCC, sensitivity, specificity, Kappa, and AUC. | The model was less accurate and likely overestimated Culex LF transmission limits. |
| 29 | Moraga P,el al, 2015^29^ | sub-Saharan Africa | UK | LF | G | Survey, literature review | true absence | R-INLA | Climate, LULC, Water, vegetation, human population | Stepwise selection using AIC | LOOCV used correlation, ME, and MAE for evaluation. | limited drivers, methods, excluded interventions, and faced data gaps, reducing map precision. |
| 30 | RACHMAWATI1R, el al, 2024^30^ | Indonesia | Indonesia | LF | G | not stated | not stated | R-INLA, QGIS​ | Elevation, geographic and demographic variables​ | Bayesian model selection using Deviance Information Criterion (DIC)​ | Bayesian inference and model Deviance Information Criterion (DIC). | Excess zeros in data, Spatial heterogeneity in case distribution, Limited environmental predictor availability​ |
| 31 | Eneanya OA,el al, 2018^31^ | Nigeria | UK | LF | ENM | Survey | true absence | R, MaxEnt | Climate, LULC, soil, topography, vegetation, and atmospheric variables. | Stepwise VIF exclusion and machine learning for variable selection. | cross-validation, ROC, and TSS scores. | data sparsity and limitations in field surveys due to conflict zones |
| 32 | Kwarteng EVS,el al,2021^32^ | Ghana | Ghana | LF | ENM | Survey, literature review | true absence | R, ArcGIS | Temperature, precipitation, LCLU, topography, water bodies, vegetation, and human population. | Stepwise selection, retaining covariates changing OR confirmed no multicollinearity via VIF. | cross-validation, AUC, sensitivity, specificity. | Data limitations, exclusion of demographic risk factors, limited sample size |
| 33 | Lindsay S W,el al, 2000^33^ | African countries | UK | LF | G | Survey, literature review | true absence | Arc/Info GIS, SPSS | Climate data. | Based on literature and known vector ecology. | varied probability cutoffs, quasi-independent validation, logistic regression diagnostics. | Spatial heterogeneity, data scarcity, model assumptions​ |
| 34 | Stanton MC,el al, 2013^34^ | Burkina Faso | UK | LF | G | Survey | true absence | R, ArcGIS | Temperature, precipitation, population, LULC, topography, soil, water, and atmosphere. | Stepwise selection based on the Akaike Information Criterion (AIC). | cross-validation, Hosmer-Lemeshow test, sensitivity, specificity, and RMSE . | Data gaps, limited socio-demographic data, need for updated environmental data |
| 35 | Mwase ET,el al,2014^35^ | Zambia | Zambia | LF | G | Case notifications | Pseudoabsence only | ArcMap, R, Stata | Temperature, precipitation, LULC, population density, water bodies, elevation, and human influence index. | Correlation matrix analysis is used to filter predictors. | AUC), specificity/sensitivity​ | Data gaps, sampling bias, uncertainty in true absence points​ |
| 36 | Slater H,el al,2013^36^ | Africa | UK | LF | G | Survey | Pseudoabsence only | R, statistical | Altitude,climate variables from WorldClim, NDVI, and population density. | Univariate logistic regression and AIC; excluded variables with Spearman’s correlation to reduce multicollinearity. | sensitivity analysis ,DIC and cross-validation. | Data inconsistencies, assumed stable transmission,challenges with complex ecology, spatial modeling, and climate uncertainties. |
| 37 | Slater H,el al,2012^37^ | Africa | UK | LF | ENM | Survey | Pseudoabsence only | Maxent, R, ArcGIS | Population density, altitude, NDVI, climatic variables. | Correlation analysis to eliminate highly correlated predictors and reduce multicollinearity in the model | quadratic discriminant analysis,AUC-ROC. | Model accuracy was limited by data gaps and inconsistent resolution. |
| 38 | Deshpande A,el al,2020^38^ | global 73 countries | USA | LF | ENM | Survey | true absence | R, ArcGIS | Environmental, socioeconomic andintervention indicators | Predictors were selected to maximize accuracy, not causality. VIF was used to remove collinear variables. | out-of-sample predictions; assessed bias, RMSE, MAE, observed–predicted correlations. | LF data was sparse and uneven, with small samples and missing vector/parasite info, increasing model uncertainty. |
| 39 | J Helen,el al,2020^39^ | Samoa | Australia | LF | G | Survey | true absence | ArcGIS, R | Elevation, slope, climate variables, NDVI, and night-time light intensity. | selected for relevance, quality, low collinearity, and Random Forest importance. | cross-validation, AUC, sensitivity, specificity, PPV, and NPV | Data were hard to collect, sampling was biased, and models varied; tailored approaches are needed. |
| 40 | Eneanya O,el al,2023^40^ | Nigeria | USA | LF, malaria | ENM | Survey | true absence | ArcGIS,R | Climatic variables and population density | used overlaying and covariate selection, followed by RF to tune the QRF model. | Root Mean Squared Error (RMSE),Pearson’s correlation coefficient, and cross-validation. | Data quality, model complexity, spatial variation, covariates, uncertainty, and limited generalization |
| 41 | Barrett C,el al, 2024^41^ | Malawi | UK | LF | G | Survey | Pseudoabsence only | R, QGIS | Climatic variables | based on literature and data availability. Antigenemia predictions were used to model clinical cases. | Comparison with reported cases​ | Not explicitly mentioned |
| 42 | Prada JM,el al, 2024^42^ | Ethiopia | UK | LF | G | Case notifications, survey | Pseudoabsence only | R, QGIS | ITN coverage and MDA history | Based on transmission modeling and historical control efforts​ | Bayesian importance sampling, validated with 2020 LF data, and quantified uncertainty to improve prediction reliability. | Historical data had uncertainty, MDA coverage was hard to verify, and geostatistical estimates may be inaccurate. |
| 43 | Stensgaard A,el al,2011^43^ | Uganda | Denmark | LF, malaria | G | Survey | true absence | OpenBUGS, R, STATA, ArcGIS | Temperature and precipitation, elevation, NDVI, and land cover. | Based on a literature review, screened by correlation, significance tests, and expert judgment. | Monte Carlo methods. | Not explicitly mentioned​ |
| 44 | Eneanya OA, el al,2019^44^ | Nigeria | UK | LF | ENM | Case search, survey, literature review | true absence | R, ArcGIS | Climate data, topography, soil, vegetation, atmospheric (aridity, humidity), development indicators (night light emissivity), | Literature review and variable importance analysis | Root Mean Squared Error (RMSE), R-squared, Pearson correlation | Spatial gaps in data, uncertainty in diagnostic comparisons, and potential influence of malaria interventions |
| 45 | Eneanya O,el al, 2021^45^ | Côte d’Ivoire | USA | Onchocerciasis | G | Survey | not stated | R, ArcGIS | Temperature and precipitation | Variance Inflation Factor (VIF) for multicollinearity assessment, variable importance ranking using BRT | internal cross-validation, RMSE, and Pearson’s correlation for evaluation. | Data limitations, lack of independent validation dataset, potential missing intervention data |
| 46 | Schmidt CA,el al,2019^46^ | Africa and Yemen | USA | Onchocerciasis | G | Survey | true absence | R software | Climate, topography, soil, proximity to water bodies, population density, MDA history. | Used VIF (threshold 3.0) to exclude collinear predictors; selected ecologically relevant covariates to capture spatial-temporal variation. | five-fold spatial cross-validation; assessed bias, MAE, RMSE, observed–predicted correlation. | Model misses some ecological factors, lacks full vector data, has reporting bias, and risks errors outside the training data. |
| 47 | Cromwell E, el al, 2021^47^ | Ethiopia | UK | Onchocerciasis | G | Survey | true absence | R ,INL, SPDE | Isothermality, precipitation seasonality, population density, river proximity, slope, and NDVI. | Variables were selected by checking multicollinearity using Pearson correlation thresholds. | 10-fold cross-validation; Pearson correlation and RMSE. | The model lacks some key data and covariates and uses outdated prevalence data that may not reflect current disease status. |
| 48 | Surakat OA,el al,2023^48^ | Nigeria | Nigeria | Onchocerciasis | ENM | Survey | Pseudoabsence only | ArcGIS,Maxent , R | Climate variables | Started with 19 variables; removed the selected 8 low-correlation variables for final model. | sensitivity analysis ROC-AUC. | Limited data and variables may reduce accuracy; MaxEnt used for presence-only, small samples. |
| 49 | Barro AS,el al,2012^49^ | Ghana and Burundi | USA | Onchocerciasis | G | Survey | true absence | ArcGIS, SAS, Microsoft Office Excel | Temperature, humidity, rainfall, elevation, land cover, and river proximity. | PCA. | (RMSE) A smaller RMSE | Limited weather data and underreporting likely led to underestimated prevalence; socioeconomic factors were excluded. |
| 50 | O’Hanlon SJ,el al,2016^50^ | west africa | UK | Onchocerciasis | G | Survey | true absence | R, ArcGIS | Temperature, precipitation, LULC, topography, water bodies, vegetation, and human population density. | Bayesian stepwise selection process based on model fit (DIC criteria) | Pearson correlation, AUC, ROC curves, prediction errors | Incomplete survey coverage, spatial variation in vector distribution, uncertainty in model estimates |
| 51 | Shrestha H,el al, 2022^51^ | Ethopia | Australia | Onchocerciasis (Onchocerca volvulus)​ | G | Survey | Pseudoabsence only | R, statistical software | Elevation, temperature, precipitation, socio-demographic, hydrological, and vegetation data | Excluded collinear variables using correlation VIF, DIC, WAIC, scores, retaining one variable per group | DDIC, WAIC, cross-validation​ | Data gaps, spatial heterogeneity, reliance on historical data​ |
| 52 | Deribe K,el al, 2019^52^ | Rwanda | Ethiopia | Podoconiosis | G | Survey | true absence | R, ArcGIS | Temperature, precipitation, topography, soil, vegetation, and water bodies. | Based on prior literature and relevance to podoconiosis risk | Not stated | Risk of underestimation due to limited case detection, missing hygiene/footwear data, and small sample size. |
| 53 | Molla YB,el al, 2014^53^ | Ethiopia | UK | Podoconiosis | G | Survey, literature review | not stated | R, ArcGIS | Soil characteristics, topography, and precipitation as key variables. | Stepwise regression model selection | Chi-square and residual deviance | Uncertainty in soil mineral measurement, difficulty in accounting for individual risk factors |
| 54 | Deribe K,el al,2023^54^ | kenya | Ethiopia | Podoconiosis | G | Survey | Pseudoabsence only | R, ArcGIS | Temperature and precipitation, topography, soil, atmospheric, and vegetation data, and distance to water bodies. | Principal Component Analysis (PCA) for dimensionality reduction, variable selection using machine learning methods | cross-validation, uncertainty assessment, AUC, TSS, and ROC analysis. | Data sparsity, absence of genetic and behavioral risk factors in the model, and underreporting due to stigma |
| 55 | Deribe K,el al, 2018^55^ | Cameroon | Ethiopia | podoconiosis | ENM | Survey | True Absence and Pseudoabsence | R, ArcGIS | Soil, climate, topography, vegetation, water proximity, and night-time lights. | based on the literature, then refined using RF and BRT. | sensitivity analysis, AUC and TSS. | Faced geographic bias, lacked individual risk data, had high unexplained variation under 1.2 km, and limited geo-referenced data. |
| 56 | Deribe K,el al,2015^56^ | Ethiopia | Ethiopia | podoconiosis | ENM | Survey | true absence | Microsoft Excel, R, STATA, ArcGIS, | Elevation, climate, vegetation, soil, water distance, LULC, population density, urban classification, and aridity index. | on relevance to podoconiosis, data availability, and low multicollinearity. | thresholds to the BRT using accuracy, sensitivity, specificity, Kappa, and AUC. | limited data accuracy, model complexity, multicollinearity, scale constraints, prediction uncertainty,limited generalizability across regions. |
| 57 | Deribe K,el al,2020^57^ | Africa | Ethiopia | podoconiosis | ENM | Survey, literature review | True Absence and Pseudoabsence | R, ArcGIS | Precipitation, LST and EVI, elevation, soil data, and water proximity. | Literature review | partial dependence plots, threshold sensitivity; AUC, TSS; spatial block cross-validation. | The model predicts suitability, not disease presence; limited surveys and missing factors like footwear use may bias results. |
| 58 | Deribe K,el al,2017^58^ | Ethiopia | Ethiopia | podoconiosis | G | Survey | true absence | R software, ArcGIS | Elevation, slope, precipitation, EVI, topsoil composition (clay and silt), and night light emissivity. | Selected predictors linked to podoconiosis, checked multicollinearity, and refined via geostatistical logistic regression. | Monte Carlo simulation, spatial residual, producing prevalence, uncertainty, and exceedance maps. | Lacked individual data and key covariates; wide confidence intervals and data gaps reduced model accuracy. |
| 59 | Hassan R,2021^59^ | Sudan | Sudan | mycetoma | ENM | Case notifications | Pseudoabsence only | ArcGIS, R | Climate, soil characteristics, livestock density, topography, vegetation. | Ecological relevance guided selection, refined by regression and ML; key predictors included aridity, water access, soil minerals, and thorny trees. | TSS, AUC, Kappa, sensitivity | Data scarcity, lack of true absence data, and biases from under-reporting or misdiagnosis limit model accuracy. |
| 60 | Samy AM,2014^60^ | Sudan, South Sudan | Egypt | mycetoma | ENM | Literature review | Pseudoabsence only | MaxEnt, Google Earth,ArcGIS | Soil characteristics (type, composition, moisture), land surface temperature (LST), vegetation density measured by NDVI, and the distribution of Acacia trees. | Selected predictors from Sudan studies, calibrated with Mycetoma Research Center data, evaluated using partial ROC and niche overlap tests. | AUC, ROC | Limited environmental data, assumptions of stable ecological niches, and calibration area choice can reduce model accuracy. |
| 61 | Hassan R,el al, 2022^61^ | Sudan | Sudan | mycetoma | G | Passive surveillance | Pseudoabsence only | R statistical | Climate, sanitation, and population density | Based on previous studies and exploratory analysis​ | Monte Carlo validation, empirical semi-variogram​ | Surveillance underreporting, geographic bias, lack of census data​ |
| 62 | Deka MA,el al,2021^62^ | Multiple countries across Latin America | USA | Tungiasis | ENM | Literature review | Pseudoabsence only | R, ArcGIS | Climate data, soil, vegetation, land cover, water proximity, livestock, and socioeconomic data. | Principal Component Analysis (PCA) | Partial ROC, AUC, Akaike Information Criterion (AICc) | Historical data gaps, ecological suitability vs. actual transmission risk, lack of real-time surveillance data |
| 63 | Hyuga A,el al, 2021^63^ | kenya | Japan | Tungiasis | G | Survey | true absence | R, QGIS | Household and environmental data including vegetation (NDVI), land cover, wetness, elevation, and soil properties | Used stepwise selection by adding covariates that changed OR >10%; VIF confirmed no collinearity. | spatial risk adjustment models with global, pointwise permutation tests for spatial association. | Data limitations, lack of socioeconomic and animal ownership data, potential underreporting due to stigma |
| 64 | Deka MA,2020^64^ | Sub-Saharan Africa | USA | Tungiasis | G | Literature review | Pseudoabsence only | R, ArcGIS | Temperature, precipitation, land cover, soil, water proximity, population, and livestock density as key covariates. | Principal Component Analysis (PCA) and stepwise selection methods applied | AUC, TSS, Cohen’s Kappa​ | Data scarcity, historical biases, limitations in pseudo-absence selection​ |
| 65 | Simpson, 2021^65^ | Continental Africa | UK | Buruli ulcer | ENM | Literature review, passive surveillance, case searches, and case notifications | Pseudoabsence only | R, MaxEnt | Climate, LULC, Topographical, Soil characteristics, Proximity to Water Bodies. | Literature review, removal of correlated predictors | AUC-ROC. | data gaps, pseudoabsence bias, and risks of overfitting and multicollinearity in modeling. |
| 66 | Campbell LP,el al, 2015^66^ | Benin | USA | Buruli ulcer | G | Passive surveillance | Pseudoabsence only | R, QGIS | Land cover data, Landsat ETM imagery, wetland, forest, and agriculture/forest classifications​ | by analyzing spatial autocorrelation and examining land cover characteristics related to disease risk | stepwise regression, DIC ,RMSPE for prediction error. | Bias due to missing environmental factors, limitations in LULC classification​ |
| 67 | Duarte-Cunha M,el al, 2016^67^ | Brazil | Brazil | Leprosy | G | Case notifications, passive surveillance | not stated | ArcGIS, R, Microsoft Excel | Climate and human population density | Stepwise selection based on Akaike Information Criterion (AIC) | AIC, Adjusted R², and Moran’s I for residual spatial autocorrelation. | aggregation of data at the neighborhood level, limited individual-level data |
| 68 | Eneanya OA.el al,2024^68^ | Chad | USA | Dracunculus medinensis | ENM | case search | Pseudoabsence only | R (biomod2) | temperature and precipitation, freshwater availability, elevation, slope of terrain, distance to water bodies, distance to nature reserves, landcover, vegetation cover, population density, nighttime light emissive, cattle distribution, food insecurity, conflict events and insecurity | used covariates known to be associated with transmission (lit review). VIF to assess multicollinearity | internal cross-validation to derive receiver operating characteristic (ROC) curve and true skill statistic [TSS] | cultural and behavioral factors not captured by models. Detection of occurrence depends on the sensitivity of the surveillance system which may be impacted by security and lower in nomadic and border populations. Does not capture impact of interventions |

1. Abbreviation:

   CL = cutaneous leishmaniasis, G = Geospatial, ENM = Ecological Niche Model, LF = Lymphatic filariasis, AUC = Area Under the Curve, AIC = Akaike Information Criterion, ROC = Receiver Operating Characteristic, ANOVA = Analysis of Variance., PCC = Proportion Correctly Classified, TSS = True Skill Statistic, RMSE = Root Mean Square Error

   PPV = Positive Predictive Value, NPV = Negative Predictive Value, WAIC = Widely Applicable Information Criterion, DIC = Deviance Information Criterion, BRT Boosted Regression Tree, LOOCV = Leave-one-out cross-validation.

   **References:**

   1. Rajabi M, Pilesjö P, Shirzadi MR, Fadaei R, Mansourian A. A spatially explicit agent-based modeling approach for the spread of Cutaneous Leishmaniasis disease in central Iran, Isfahan. *Env Model Softw*. 2016;82(C):330-346. doi:10.1016/j.envsoft.2016.04.006

   2. Purse BV, Masante D, Golding N, et al. How will climate change pathways and mitigation options alter incidence of vector-borne diseases? A framework for leishmaniasis in South and Meso-America. *PLOS ONE*. 2017;12(10):e0183583. doi:10.1371/journal.pone.0183583

   3. Karagiannis-Voules DA, Scholte RGC, Guimarães LH, Utzinger J, Vounatsou P. Bayesian Geostatistical Modeling of Leishmaniasis Incidence in Brazil. *PLoS Negl Trop Dis*. 2013;7(5):e2213. doi:10.1371/journal.pntd.0002213

   4. Wijerathna T, Wickramasinghe K, Gunathilaka N, Perera A, Bandara S. The epidemiological trend of cutaneous leishmaniasis in Kegalle district, Sri Lanka: A newly established disease focus and assessment of bioclimatic suitability for disease establishment using ecological niche modelling. *Acta Trop*. 2023;237:106719. doi:10.1016/j.actatropica.2022.106719

   5. King R, Campbell-Lendrum D, Davies C. Predicting Geographic Variation in Cutaneous Leishmaniasis, Colombia. *Emerg Infect Dis*. 2004;10:598-607. doi:10.3201/eid1004.030241

   6. Ullah W, Yen TY, Niaz S, et al. Distribution and Risk of Cutaneous Leishmaniasis in Khyber Pakhtunkhwa, Pakistan. *Trop Med Infect Dis*. 2023;8(2):128. doi:10.3390/tropicalmed8020128

   7. Zeb I, Qureshi N, Shaheen N, et al. Spatiotemporal patterns of Cutaneous Leishmaniasis in the District Upper and Lower Dir, Khyber Pakhtunkhwa, Pakistan: A GIS-based Spatial Approaches. *Acta Trop*. Published online February 12, 2021:105861. doi:10.1016/j.actatropica.2021.105861

   8. Shabanpour N, Razavi-Termeh SV, Sadeghi-Niaraki A, Choi SM, Abuhmed T. Integration of machine learning algorithms and GIS-based approaches to cutaneous leishmaniasis prevalence risk mapping. *Int J Appl Earth Obs Geoinformation*. 2022;112:102854. doi:10.1016/j.jag.2022.102854

   9. Jagadesh S, Combe M, Ginouvès M, et al. Spatial variations in Leishmaniasis: A biogeographic approach to mapping the distribution of Leishmania species. *One Health*. 2021;13:100307. doi:10.1016/j.onehlt.2021.100307

   10. Artun O. Ecological niche modeling for the prediction of cutaneous leishmaniasis epidemiology in current and projected future in Adana, Turkey. *J Vector Borne Dis*. 2019;56:127-133. doi:10.4103/0972-9062.263726

   11. Adegboye OA, Adegboye M. Spatially Correlated Time Series and Ecological Niche Analysis of Cutaneous Leishmaniasis in Afghanistan. *Int J Environ Res Public Health*. 2017;14(3):309. doi:10.3390/ijerph14030309

   12. Andreo V, Rosa J, Ramos K, Salomón OD. Ecological characterization of a cutaneous leishmaniasis outbreak through remotely sensed land cover changes. *Geospatial Health*. 2022;17(1). doi:10.4081/gh.2022.1033

   13. Pérez-Flórez M, Ocampo CB, Valderrama-Ardila C, Alexander N. Spatial modeling of cutaneous leishmaniasis in the Andean region of Colombia. *Mem Inst Oswaldo Cruz*. 2016;111(7):433-442. doi:10.1590/0074-02760160074

   14. Seid A, Gadisa E, Tsegaw T, et al. Risk map for cutaneous leishmaniasis in Ethiopia based on environmental factors as revealed by geographical information systems and statistics. *Geospatial Health*. 2014;8(2):377-387. doi:10.4081/gh.2014.27

   15. Chavy A, Ferreira Dales Nava A, Luz SLB, et al. Ecological niche modelling for predicting the risk of cutaneous leishmaniasis in the Neotropical moist forest biome. *PLoS Negl Trop Dis*. 2019;13(8):e0007629. doi:10.1371/journal.pntd.0007629

   16. B C, S C, B M, et al. Ecological Niche Modeling for the Prediction of the Geographic Distribution of Cutaneous Leishmaniasis in Tunisia. *Am J Trop Med Hyg*. 2016;94(4). doi:10.4269/ajtmh.15-0345

   17. Artun O, Kavur H. Determination of the Future Projection of Cutaneous Leishmaniasis Using Ecological Niche Modeling: Diyarbakır Province. *J Inst Sci Technol*. 2019;9:1253-1261. doi:10.21597/jist.519090

   18. Artun O, Kavur H. Prediction of Cutaneous Leishmaniasis Epidemiology in Mersin Using Ecological Niche Modeling. *Turk Parazitolojii Derg*. 2018;42(3):191-195. doi:10.5152/tpd.2018.5924

   19. Artun O, Kavur H. A Comparison of Bioclimatic Factors Effect For Cutaneous Leishmaniasis. Published online September 9, 2019.

   20. Amro A, Al-Dwibe H, Gashout A, et al. Spatiotemporal and molecular epidemiology of cutaneous leishmaniasis in Libya. *PLoS Negl Trop Dis*. 2017;11(9):e0005873. doi:10.1371/journal.pntd.0005873

   21. Meneguzzi VC, Santos CBD, Leite GR, Fux B, Falqueto A. Environmental Niche Modelling of Phlebotomine Sand Flies and Cutaneous Leishmaniasis Identifies Lutzomyia intermedia as the Main Vector Species in Southeastern Brazil. *PloS One*. 2016;11(10):e0164580. doi:10.1371/journal.pone.0164580

   22. Trájer AJ, Grmasha RA. The potential effects of climate change on the climatic suitability patterns of the Western Asian vectors and parasites of cutaneous leishmaniasis in the mid- and late twenty-first century. *Theor Appl Climatol*. 2024;155(3):1897-1914. doi:10.1007/s00704-023-04726-4

   23. Pigott DM, Bhatt S, Golding N, et al. Global distribution maps of the leishmaniases. *eLife*. 2014;3:e02851. doi:10.7554/eLife.02851

   24. Moo-Llanes DA. [Current and future ecological niche of Leishmaniasis (Kinetoplastida: Trypanosomatidae) in the Neotropical region]. *Rev Biol Trop*. 2016;64(3):1237-1245.

   25. Ovalle-Bracho C, Londoño-Barbosa D, Salgado-Almario J, González C. Evaluating the spatial distribution of Leishmania parasites in Colombia from clinical samples and human isolates (1999 to 2016). *PloS One*. 2019;14(3):e0214124. doi:10.1371/journal.pone.0214124

   26. Navi Z, Salahi-Moghaddam A, Habibi-Nokhandan M, Mohebali M, Hajjaran H, Màrius Vicent F i F. A Geomedical Survey: Is There an Association Between Climatic Conditions and Leishmania Species Distribution in Iran During the Years 1999-2021? *Acta Parasitol*. 2024;69. doi:10.1007/s11686-024-00811-4

   27. Cadavid Restrepo AM, Martin BM, Fuimaono S, Clements ACA, Graves PM, Lau CL. Spatial predictive risk mapping of lymphatic filariasis residual hotspots in American Samoa using demographic and environmental factors. *PLoS Negl Trop Dis*. 2023;17(7):e0010840. doi:10.1371/journal.pntd.0010840

   28. Cano J, Rebollo MP, Golding N, et al. The global distribution and transmission limits of lymphatic filariasis: past and present. *Parasit Vectors*. 2014;7:466. doi:10.1186/s13071-014-0466-x

   29. Moraga P, Cano J, Baggaley RF, et al. Modelling the distribution and transmission intensity of lymphatic filariasis in sub-Saharan Africa prior to scaling up interventions: integrated use of geostatistical and mathematical modelling. *Parasit Vectors*. 2015;8(1):560. doi:10.1186/s13071-015-1166-x

   30. Bayesian spatial hierarchical mixture models for excess zeros data: review and application to female lymphatic filariasis cases. *Commun Math Biol Neurosci*. Published online 2024. doi:10.28919/cmbn/8428

   31. Eneanya OA, Cano J, Dorigatti I, et al. Environmental suitability for lymphatic filariasis in Nigeria. *Parasit Vectors*. 2018;11:513. doi:10.1186/s13071-018-3097-9

   32. Kwarteng EVS, Andam-Akorful SA, Kwarteng A, et al. Spatial variation in lymphatic filariasis risk factors of hotspot zones in Ghana. *BMC Public Health*. 2021;21(1):230. doi:10.1186/s12889-021-10234-9

   33. Lindsay SW, Thomas CJ. Mapping and estimating the population at risk from lymphatic filariasis in Africa. *Trans R Soc Trop Med Hyg*. 2000;94(1):37-45. doi:10.1016/S0035-9203(00)90431-0

   34. Stanton MC, Molyneux DH, Kyelem D, Bougma RW, Koudou BG, Kelly-Hope LA. Baseline drivers of lymphatic filariasis in Burkina Faso. *Geospatial Health*. 2013;8(1):159-173. doi:10.4081/gh.2013.63

   35. Mwase ET, Stensgaard AS, Nsakashalo-Senkwe M, et al. Mapping the geographical distribution of lymphatic filariasis in Zambia. *PLoS Negl Trop Dis*. 2014;8(2):e2714. doi:10.1371/journal.pntd.0002714

   36. Slater H, Michael E. Mapping, Bayesian Geostatistical Analysis and Spatial Prediction of Lymphatic Filariasis Prevalence in Africa. *PLoS ONE*. 2013;8(8):e71574. doi:10.1371/journal.pone.0071574

   37. Slater H, Michael E. Predicting the current and future potential distributions of lymphatic filariasis in Africa using maximum entropy ecological niche modelling. *PloS One*. 2012;7(2):e32202. doi:10.1371/journal.pone.0032202

   38. Deshpande A, Miller-Petrie MK, Johnson KB, et al. The global distribution of lymphatic filariasis, 2000–18: a geospatial analysis. *Lancet Glob Health*. 2020;8(9):e1186-e1194. doi:10.1016/S2214-109X(20)30286-2

   39. Mayfield HJ, Sturrock H, Arnold BF, et al. Supporting elimination of lymphatic filariasis in Samoa by predicting locations of residual infection using machine learning and geostatistics. *Sci Rep*. 2020;10:20570. doi:10.1038/s41598-020-77519-8

   40. Eneanya O, Reimer L, Fischer P, Weil G. Geospatial modelling of lymphatic filariasis and malaria co-endemicity in Nigeria. *Int Health*. 2023;15. doi:10.1093/inthealth/ihad029

   41. Barrett C, Chiphwanya J, Mkwanda S, et al. The national distribution of lymphatic filariasis cases in Malawi using patient mapping and geostatistical modelling. *PLoS Negl Trop Dis*. 2024;18(3):e0012056. doi:10.1371/journal.pntd.0012056

   42. Prada JM, Touloupou P, Kebede B, et al. Subnational Projections of Lymphatic Filariasis Elimination Targets in Ethiopia to Support National Level Policy. *Clin Infect Dis*. 2024;78(Supplement_2):S117-S125. doi:10.1093/cid/ciae072

   43. Stensgaard AS, Vounatsou P, Onapa AW, et al. Bayesian geostatistical modelling of malaria and lymphatic filariasis infections in Uganda: predictors of risk and geographical patterns of co-endemicity. *Malar J*. 2011;10:298. doi:10.1186/1475-2875-10-298

   44. Eneanya OA, Fronterre C, Anagbogu I, et al. Mapping the baseline prevalence of lymphatic filariasis across Nigeria. *Parasit Vectors*. 2019;12(1):440. doi:10.1186/s13071-019-3682-6

   45. Eneanya O, Koudou B, Aboulaye M, et al. Progress towards onchocerciasis elimination in Côte d’Ivoire: A geospatial modelling study. *PLoS Negl Trop Dis*. 2021;15:e0009091. doi:10.1371/journal.pntd.0009091

   46. Schmidt CA, Cromwell EA, Hill E, et al. The prevalence of onchocerciasis in Africa and Yemen, 2000–2018: a geospatial analysis. *BMC Med*. 2022;20(1):293. doi:10.1186/s12916-022-02486-y

   47. Cromwell E, Al-Aly Z, al et. Predicting the environmental suitability for onchocerciasis in Africa as an aid to elimination planning. *PLoS Negl Trop Dis*. 2021;15(7):e0008824. doi:10.1371/journal.pntd.0008824

   48. Surakat OA, Babalola AS, Adeleke MA, Adeogun AO, Idowu OA, Sam-Wobo SO. Geospatial distribution and predictive modeling of onchocerciasis in Ogun State, Nigeria. *PLOS ONE*. 2023;18(3):e0281624. doi:10.1371/journal.pone.0281624

   49. Barro AS, Oyana TJ. Predictive and epidemiologic modeling of the spatial risk of human onchocerciasis using biophysical factors: A case study of Ghana and Burundi. *Spat Spatio-Temporal Epidemiol*. 2012;3(4):273-285. doi:10.1016/j.sste.2012.08.001

   50. O’Hanlon SJ, Slater HC, Cheke RA, et al. Model-Based Geostatistical Mapping of the Prevalence of Onchocerca volvulus in West Africa. *PLoS Negl Trop Dis*. 2016;10(1):e0004328. doi:10.1371/journal.pntd.0004328

   51. Shrestha H, McCulloch K, Hedtke SM, Grant WN. Geospatial modeling of pre-intervention nodule prevalence of Onchocerca volvulus in Ethiopia as an aid to onchocerciasis elimination. *PLoS Negl Trop Dis*. 2022;16(7):e0010620. doi:10.1371/journal.pntd.0010620

   52. Deribe K, Mbituyumuremyi A, Cano J, et al. Geographical distribution and prevalence of podoconiosis in Rwanda: a cross-sectional country-wide survey. *Lancet Glob Health*. 2019;7(5):e671-e680. doi:10.1016/S2214-109X(19)30072-5

   53. Molla YB, Wardrop NA, Le Blond JS, et al. Modelling environmental factors correlated with podoconiosis: a geospatial study of non-filarial elephantiasis. *Int J Health Geogr*. 2014;13:24. doi:10.1186/1476-072X-13-24

   54. Deribe K, Sultani H, Okoyo C, et al. Geostatistical modelling of the distribution, risk and burden of podoconiosis in Kenya. *Trans R Soc Trop Med Hyg*. 2022;117:1-11. doi:10.1093/trstmh/trac092

   55. Deribe K, Cano J, Njouendou AJ, et al. Predicted distribution and burden of podoconiosis in Cameroon. *BMJ Glob Health*. 2018;3(3):e000730. doi:10.1136/bmjgh-2018-000730

   56. Deribe K, Cano J, Newport MJ, et al. Mapping and Modelling the Geographical Distribution and Environmental Limits of Podoconiosis in Ethiopia. *PLoS Negl Trop Dis*. 2015;9(7):e0003946. doi:10.1371/journal.pntd.0003946

   57. Deribe K, Simpson H, Pullan RL, et al. Predicting the environmental suitability and population at risk of podoconiosis in Africa. *PLoS Negl Trop Dis*. 2020;14(8):e0008616. doi:10.1371/journal.pntd.0008616

   58. Deribe K, Cano J, Giorgi E, et al. Estimating the number of cases of podoconiosis in Ethiopia using geostatistical methods. *Wellcome Open Res*. 2017;2:78. doi:10.12688/wellcomeopenres.12483.2

   59. Hassan R, Simpson H, Cano J, et al. Modelling the spatial distribution of mycetoma in Sudan. *Trans R Soc Trop Med Hyg*. 2021;115(10):1144-1152. doi:10.1093/trstmh/trab076

   60. Sande W, Fahal A, Peterson A. Mapping the Potential Risk of Mycetoma Infection in Sudan and South Sudan Using Ecological Niche Modeling. *PLoS Negl Trop Dis*. 2014;8. doi:10.1371/journal.pntd.0003250

   61. Hassan R, Cano J, Fronterre C, et al. Estimating the burden of mycetoma in Sudan for the period 1991-2018 using a model-based geostatistical approach. *PLoS Negl Trop Dis*. 2022;16(10):e0010795. doi:10.1371/journal.pntd.0010795

   62. Deka MA, Heukelbach J. Distribution of tungiasis in latin America: Identification of areas for potential disease transmission using an ecological niche model. *Lancet Reg Health Am*. 2022;5:100080. doi:10.1016/j.lana.2021.100080

   63. Hyuga A, Larson PS, Ndemwa M, et al. Environmental and Household-Based Spatial Risks for Tungiasis in an Endemic Area of Coastal Kenya. *Trop Med Infect Dis*. 2021;7(1):2. doi:10.3390/tropicalmed7010002

   64. Deka MA. Mapping the Geographic Distribution of Tungiasis in Sub-Saharan Africa. *Trop Med Infect Dis*. 2020;5(3):122. doi:10.3390/tropicalmed5030122

   65. Simpson H, Tabah EN, Phillips RO, et al. Mapping suitability for Buruli ulcer at fine spatial scales across Africa: A modelling study. *PLoS Negl Trop Dis*. 2021;15(3):e0009157. doi:10.1371/journal.pntd.0009157

   66. Campbell LP, Finley AO, Benbow ME, et al. Spatial Analysis of Anthropogenic Landscape Disturbance and Buruli Ulcer Disease in Benin. *PLoS Negl Trop Dis*. 2015;9(10):e0004123. doi:10.1371/journal.pntd.0004123

   67. Duarte-Cunha M, Almeida AS de, Cunha GM da, Souza-Santos R. Geographic weighted regression: applicability to epidemiological studies of leprosy. *Rev Soc Bras Med Trop*. 2016;49(1):74-82. doi:10.1590/0037-8682-0307-2015

   68. Eneanya OA, Delea MG, Cano J, et al. Predicting the Environmental Suitability and Identifying Climate and Sociodemographic Correlates of Guinea Worm (Dracunculus medinensis) in Chad. *Am J Trop Med Hyg*. 2024;111(3 Suppl):26-35. doi:10.4269/ajtmh.23-0681 [↑](#endnote-ref-1)
