# Supplementary material for: Modelling approaches for predicting the distribution of skin NTDs: A systematic review
Source: PLoS Negl Trop Dis. 2026 May 4;20(5):e0013662. doi: 10.1371/journal.pntd.0013662 (PMC13155686; doi:10.1371/journal.pntd.0013662)
Supplement: S3 File — Risk of bias and quality assessments for the 68 included studies. (DOCX) [file pntd.0013662.s003.docx]

| No | Study | Were the criteria for inclusion in the sample clearly defined? | Were the study area and population clearly defined and described in detail? | Were the participants representative of the population of interest? | Was the exposure location measured in a valid and reliable way? | Were environmental covariates handled in a valid and reliable way? | Were objective, standard criteria used to define cases? | Was the selection of background / pseudoabsence data fully reported and appropriate? | Was sampling bias addressed? | Was spatial autocorrelation addressed? | Was appropriate statistical analysis used? | Overall RoB |
| --- | --- | --- | --- | --- | --- | --- | --- | --- | --- | --- | --- | --- |
| 1 | A spatially explicit agent-based modeling approach for the spread of Cutaneous Leishmaniasis disease in central Iran, Isfahan | No | Yes | Unclear | Unclear | Partly | Unclear | Not applicable | No | Yes | Yes | Moderate |
| 2 | How will climate change pathways and mitigation options alter incidence of vector-borne diseases? A framework for leishmaniasis in South and Meso-America | Yes | Yes | Unclear | No | Yes | Unclear | Yes | Yes | Partly | Yes | Moderate |
| 3 | Bayesian Geostatistical Modeling of Leishmaniasis Incidence in Brazil | Partly | Yes | No | No | Partly | Unclear | Not applicable | No | Yes | Yes | High |
| 4 | The epidemiological trend of cutaneous leishmaniasis in Kegalle district, Sri Lanka: A newly established disease focus and assessment of bioclimatic suitability for disease establishment using ecological niche modelling | Partly | Yes | No | Yes | Partly | Unclear | Yes | Yes | No | Partly | Moderate |
| 5 | predicting geographic variation in cutaneous leishmaniasis colombia | Partly | Yes | No | Partly | Yes | Unclear | Not applicable | No | No | Yes | Moderate |
| 6 | Distribution and Risk of Cutaneous Leishmaniasis in Khyber Pakhtunkhwa, Pakistan | Partly | Yes | No | No | Yes | Unclear | Not applicable | No | Unclear | No | High |
| 7 | Spatiotemporal patterns of cutaneous leishmaniasis in the district upper and lower Dir, Khyber Pakhtunkhwa, Pakistan: A GIS-based spatial approaches | Yes | Yes | No | Partly | Not applicable | Yes | Not applicable | Not applicable | Yes | Yes | Moderate |
| 8 | Integration of machine learning algorithms and GIS-based approaches to cutaneous leishmaniasis prevalence risk mapping. | Yes | Yes | No | Unclear | Yes | Unclear | Not applicable | No | Yes | Yes | Moderate |
| 9 | Spatial variations in Leishmaniasis: A biogeographic approach to mapping the distribution of Leishmania species. | Yes | Yes | No | Yes | Yes | Unclear | Yes | Yes | No | Unclear | Moderate |
| 10 | Ecological niche modeling for the prediction of cutaneous leishmaniasis epidemiology in current and projected future in Adana, Turkey. | No | Yes | No | Unclear | Yes | Unclear | Yes | No | No | Unclear | High |
| 11 | Ecological niche modelling for predicting the risk of cutaneous leishmaniasis in the Neotropical moist forest biome | Yes | Yes | No | Unclear | Yes | Unclear | Yes | Partly | Yes | Yes | Moderate |
| 12 | Spatially Correlated Time Series and Ecological Niche Analysis of Cutaneous Leishmaniasis in Afghanistan. | Yes | Yes | No | No | Yes | Yes | Unclear | No | No | No | High |
| 13 | Ecological characterization of a cutaneous leishmaniasis outbreak through remotely sensed land cover changes | Yes | Yes | Unclear | Yes | Yes | Unclear | Yes | Yes | Yes | Yes | Low |
| 14 | Spatial modeling of cutaneous leishmaniasis in the Andean region of Colombia. | Yes | Yes | No | No | Yes | Unclear | Not applicable | No | Yes | Yes | High |
| 15 | Risk map for cutaneous leishmaniasis in Ethiopia based on environmental factors as revealed by geographical information systems and statistics. | Yes | Yes | No | Partly | Yes | Unclear | Not applicable | No | No | Yes | High |
| 16 | Ecological Niche Modeling for the Prediction of the Geographic Distribution of Cutaneous Leishmaniasis in Tunisia. | Yes | Yes | No | Yes | Yes | Yes | Yes | Yes | Partly | Yes | Moderate |
| 17 | determination of the future projection of cutaneous leishmaniasis using ecological niche modeling diyarbakir province turkish | Yes | Yes | No | Unclear | No | Unclear | Yes | No | No | Unclear | High |
| 18 | Prediction of Cutaneous Leishmaniasis Epidemiology in Mersin Using Ecological Niche Modeling. | Yes | Yes | No | Unclear | Unclear | Unclear | Yes | No | No | Yes | High |
| 19 | comparison of bioclimatic factors effect for cutaneous leishmaniasis current status between eastern mediterranean region and tigris basin of turkey by using ecological niche modeling | Yes | Yes | No | Unclear | Unclear | Unclear | Yes | No | No | Yes | High |
| 20 | Spatiotemporal and molecular epidemiology of cutaneous leishmaniasis in Libya. | Yes | Yes | No | Unclear | Partly | Yes | No | No | No | Yes | High |
| 21 | Environmental Niche Modelling of Phlebotomine Sand Flies and Cutaneous Leishmaniasis Identifies Lutzomyia intermedia as the Main Vector Species in Southeastern Brazil. | Yes | Yes | No | Yes | Partly | Unclear | Yes | No | No | Partly | High |
| 22 | The potential effects of climate change on the climatic suitability patterns of the Western Asian vectors and parasites of cutaneous leishmaniasis in the mid- and late twenty-first century. | Yes | Yes | No | Unclear | Yes | Unclear | Not applicable | Unclear | No | Partly | Moderate |
| 23 | Global distribution maps of the leishmaniases. | Yes | Yes | No | No | Yes | Unclear | Yes | Partly | No | Yes | High |
| 24 | Current and future ecological niche of Leishmaniasis (Kinetoplastida: Trypanosomatidae) in the Neotropical region]. | Yes | Yes | No | Unclear | Partly | Unclear | No | No | No | Yes | High |
| 25 | Evaluating the spatial distribution of Leishmania parasites in Colombia from clinical samples and human isolates (1999 to 2016). | Yes | Unclear | No | Partly | Yes | Yes | Unclear | Yes | Partly | Yes | Moderate |
| 26 | A Geomedical Survey: Is There an Association Between Climatic Conditions and Leishmania Species Distribution in Iran During the Years 1999-2021? | Partly | Yes | No | No | Not applicable | Partly | Not applicable | No | No | Partly | High |
| 27 | Spatial predictive risk mapping of lymphatic filariasis residual hotspots in American Samoa using demographic and environmental factors. | Yes | Yes | Yes | Yes | Yes | Yes | Not applicable | Not applicable | Yes | Yes | Low |
| 28 | The global distribution and transmission limits of lymphatic filariasis: past and present. | Yes | Yes | Partly | Partly | Yes | Unclear | Partly | Not applicable | No | Yes | Moderate |
| 29 | Modelling the distribution and transmission intensity of lymphatic filariasis in sub-Saharan Africa prior to scaling up interventions: integrated use of geostatistical and mathematical modelling. | Partly | Yes | Partly | Partly | Partly | Partly | Not applicable | Not applicable | Yes | Yes | Low |
| 30 | Bayesian spatial hierarchical mixture models for excess zeros data: review and application to female lymphatic filariasis cases. | Partly | Yes | No | No | Partly | Unclear | Not applicable | No | Yes | Yes | High |
| 31 | Environmental suitability for lymphatic filariasis in Nigeria | Partly | Yes | Yes | Partly | Yes | Yes | Not applicable | Not applicable | No | Yes | Moderate |
| 32 | Spatial variation in lymphatic filariasis risk factors of hotspot zones in Ghana. | Partly | Yes | Unclear | Partly | Yes | Yes | Not applicable | Not applicable | No | Yes | Moderate |
| 33 | Mapping and estimating the population at risk from lymphatic filariasis in Africa. | Partly | Yes | No | Partly | Yes | Partly | Not applicable | No | No | Yes | Moderate |
| 34 | Baseline drivers of lymphatic filariasis in Burkina Faso. | Yes | Yes | Yes | Yes | Yes | Yes | Not applicable | No | No | Yes | Moderate |
| 35 | Mapping the geographical distribution of lymphatic filariasis in Zambia. | Partly | Yes | Partly | Yes | Yes | Yes | Yes | Not applicable | No | Yes | Moderate |
| 36 | Mapping, Bayesian Geostatistical Analysis and Spatial Prediction of Lymphatic Filariasis Prevalence in Africa. | Yes | Yes | Partly | Partly | Partly | Yes | Not applicable | Not applicable | Yes | Yes | Low |
| 37 | Predicting the current and future potential distributions of lymphatic filariasis in Africa using maximum entropy ecological niche modelling | Yes | Yes | Partly | Partly | Partly | Yes | Partly | Not applicable | No | Yes | Moderate |
| 38 | The global distribution of lymphatic filariasis, 2000–18: a geospatial analysis. | Yes | Yes | Partly | Partly | Partly | Partly | Not applicable | Not applicable | Yes | Yes | Low |
| 39 | Supporting elimination of lymphatic filariasis in Samoa by predicting locations of residual infection using machine learning and geostatistics. | Yes | Yes | Yes | Yes | Yes | Yes | Not applicable | Not applicable | Yes | Yes | Low |
| 40 | Geospatial modelling of lymphatic filariasis and malaria co-endemicity in Nigeria. | Yes | Yes | Unclear | Partly | Yes | Yes | Not applicable | Not applicable | Yes | Yes | Low |
| 41 | The national distribution of lymphatic filariasis cases in Malawi using patient mapping and geostatistical modelling. | Yes | Yes | Partly | Partly | Yes | Partly | Not applicable | Not applicable | Yes | Yes | Low |
| 42 | Subnational Projections of Lymphatic Filariasis Elimination Targets in Ethiopia to Support National Level Policy | Yes | Yes | Yes | No | Yes | Yes | Not applicable | Not applicable | Yes | Yes | Moderate |
| 43 | Bayesian geostatistical modelling of malaria and lymphatic filariasis infections in Uganda: predictors of risk and geographical patterns of co-endemicity. Malar | Yes | Yes | Yes | Unclear | Partly | Yes | Not applicable | Not applicable | Yes | Yes | Low |
| 44 | Mapping the baseline prevalence of lymphatic filariasis across Nigeria. | Partly | Yes | Yes | Partly | Partly | Partly | Not applicable | Not applicable | Yes | Yes | Low |
| 45 | Progress towards onchocerciasis elimination in Côte d’Ivoire: A geospatial modelling study. | Yes | Yes | Partly | Unclear | Yes | Yes | Not applicable | Not applicable | Partly | Yes | Low |
| 46 | The prevalence of onchocerciasis in Africa and Yemen, 2000–2018: a geospatial analysis. | Partly | Yes | Partly | Partly | Partly | Partly | Not applicable | Not applicable | Yes | Yes | Low |
| 47 | Predicting the environmental suitability for onchocerciasis in Africa as an aid to elimination planning. | Yes | Yes | Partly | Partly | Partly | Yes | Yes | No | No | Yes | Moderate |
| 48 | Geospatial distribution and predictive modeling of onchocerciasis in Ogun State, Nigeria. | Partly | Partly | Yes | Unclear | Yes | Partly | Unclear | Not applicable | Yes | Yes | Low |
| 49 | Predictive and epidemiologic modeling of the spatial risk of human onchocerciasis using biophysical factors: A case study of Ghana and Burundi. | Partly | Yes | Partly | Unclear | Yes | Partly | Not applicable | Not applicable | Yes | Yes | Low |
| 50 | Model-Based Geostatistical Mapping of the Prevalence of Onchocerca volvulus in West Africa | Yes | Yes | Partly | Partly | Yes | Partly | Not applicable | Not applicable | Yes | Yes | Low |
| 51 | Geospatial modeling of pre-intervention nodule prevalence of Onchocerca volvulus in Ethiopia as an aid to onchocerciasis elimination. | Yes | Yes | Partly | Partly | Partly | Yes | Not applicable | Not applicable | Yes | Yes | Low |
| 52 | Geographical distribution and prevalence of podoconiosis in Rwanda: a cross-sectional country-wide survey. | Yes | Yes | Yes | Yes | Partly | Yes | Not applicable | Not applicable | Partly | Unclear | Low |
| 53 | Modelling environmental factors correlated with podoconiosis: a geospatial study of non-filarial elephantiasis. | Unclear | Partly | Partly | Yes | Partly | Partly | Not applicable | Not applicable | Partly | Yes | Low |
| 54 | Geostatistical modelling of the distribution, risk and burden of podoconiosis in Kenya. | Yes | Yes | Partly | Partly | Partly | Partly | Not applicable | Not applicable | Yes | Partly | Low |
| 55 | Predicted distribution and burden of podoconiosis in Cameroon. | Yes | Yes | Yes | Yes | Yes | Yes | Yes | Yes | Yes | Partly | Low |
| 56 | Mapping and Modelling the Geographical Distribution and Environmental Limits of Podoconiosis in Ethiopia. | Partly | Yes | Yes | Yes | Partly | No | Not applicable | No | No | Yes | Moderate |
| 57 | Predicting the environmental suitability and population at risk of podoconiosis in Africa. | Yes | Yes | Partly | Partly | Partly | Yes | Yes | No | No | Partly | Moderate |
| 58 | Estimating the number of cases of podoconiosis in Ethiopia using geostatistical methods. | Yes | Yes | Yes | Yes | Partly | Yes | Not applicable | Not applicable | Yes | Yes | Low |
| 59 | Modelling the spatial distribution of mycetoma in Sudan. | Yes | Yes | No | Partly | Partly | Yes | Yes | No | Unclear | Yes | Moderate |
| 60 | Mapping the Potential Risk of Mycetoma Infection in Sudan and South Sudan Using Ecological Niche Modeling. | Yes | Partly | No | Partly | Partly | Unclear | No | No | No | Partly | High |
| 61 | Estimating the burden of mycetoma in Sudan for the period 1991-2018 using a model-based geostatistical approach. | Yes | Yes | No | Partly | Partly | Unclear | Not applicable | No | Yes | Partly | Moderate |
| 62 | Distribution of tungiasis in latin America: Identification of areas for potential disease transmission using an ecological niche model. | Yes | Yes | No | Partly | Unclear | Unclear | No | No | No | Partly | High |
| 63 | Environmental and Household-Based Spatial Risks for Tungiasis in an Endemic Area of Coastal Kenya. | Yes | Yes | Yes | Yes | Yes | Yes | Not applicable | Not applicable | Yes | Yes | Low |
| 64 | Mapping the Geographic Distribution of Tungiasis in Sub-Saharan Africa. | Yes | Yes | No | Partly | Partly | Unclear | Yes | No | No | Yes | Moderate |
| 65 | Mapping suitability for Buruli ulcer at fine spatial scales across Africa: A modelling study. | Yes | Yes | No | Partly | Partly | Yes | Yes | Yes | Unclear | Yes | Moderate |
| 66 | Spatial Analysis of Anthropogenic Landscape Disturbance and Buruli Ulcer Disease in Benin. | Yes | Yes | Unclear | Partly | Yes | Yes | Not applicable | Not applicable | Yes | Yes | Low |
| 67 | Geographic weighted regression: applicability to epidemiological studies of leprosy. | Yes | Yes | No | No | Unclear | Yes | Not applicable | No | Yes | Yes | High |
| 68 | Predicting the Environmental Suitability and Identifying Climate and Sociodemographic Correlates of Guinea Worm (Dracunculus medinensis) in Chad. | Partly | Unclear | Partly | No | Partly | Yes | Yes | No | No | Yes | High |
