# Supplementary material for: Modelling approaches for predicting the distribution of skin NTDs: A systematic review
Source: PLoS Negl Trop Dis. 2026 May 4;20(5):e0013662. doi: 10.1371/journal.pntd.0013662 (PMC13155686; doi:10.1371/journal.pntd.0013662)
Supplement: S4 File — Completed PRISMA 2020 checklist indicating where each reporting item is addressed in the manuscript. (DOCX) [file pntd.0013662.s004.docx]

| **Section and Topic** | **Item #** | **Checklist item** | **Location where item is reported** |
| --- | --- | --- | --- |
| **TITLE** | | |  |
| Title | 1 | Identify the report as a systematic review. | Title page (p.1): Identified as 'A Systematic Review' in title. |
| **ABSTRACT** | | |  |
| Abstract | 2 | See the PRISMA 2020 for Abstracts checklist. | Abstract (p.3-4): Structured abstract including Background, Methods, Results, Conclusions. |
| **INTRODUCTION** | | |  |
| Rationale | 3 | Describe the rationale for the review in the context of existing knowledge. | Introduction (pp.5–8): Rationale discussed burden of skin NTDs, importance of predictive modelling, data limitations. |
| Objectives | 4 | Provide an explicit statement of the objective(s) or question(s) the review addresses. | Introduction (p.8): Objective clearly stated describe, compare, evaluate modelling approaches for skin NTDs. |
| **METHODS** | | |  |
| Eligibility criteria | 5 | Specify the inclusion and exclusion criteria for the review and how studies were grouped for the syntheses. | Methods (pp.10–11, Table 2): Inclusion/exclusion criteria for diseases, data, modelling focus, spatial scale, publication type. |
| Information sources | 6 | Specify all databases, registers, websites, organisations, reference lists and other sources searched or consulted to identify studies. Specify the date when each source was last searched or consulted. | Methods (pp.8-9): PubMed, Ovid, Web of Science searched; updated Sept 2024; references of key publications screened. |
| Search strategy | 7 | Present the full search strategies for all databases, registers and websites, including any filters and limits used. | Methods (p 8, Table 1): **Search strategy:** PubMed, Ovid, and Web of Science (2000–Sept 2024); terms on skin NTDs, prediction, and modelling (see Table 1); limited to English, French, Portuguese, Spanish, Turkish; duplicates removed in Rayyan; references screened. |
| Selection process | 8 | Specify the methods used to decide whether a study met the inclusion criteria of the review, including how many reviewers screened each record and each report retrieved, whether they worked independently, and if applicable, details of automation tools used in the process. | Methods (pp.11-12): Screening by two independent reviewers using Rayyan, disagreements resolved by third reviewer. |
| Data collection process | 9 | Specify the methods used to collect data from reports, including how many reviewers collected data from each report, whether they worked independently, any processes for obtaining or confirming data from study investigators, and if applicable, details of automation tools used in the process. | Methods (p.11-12): Data extracted independently by two reviewers into Excel; multiple predefined fields; translated non-English using Google Translate. |
| Data items | 10a | List and define all outcomes for which data were sought. Specify whether all results that were compatible with each outcome domain in each study were sought (e.g. for all measures, time points, analyses), and if not, the methods used to decide which results to collect. | Methods (pp.11-12): We extracted outcomes on disease, region, country of lead author’s institutional affiliation, data sources, model type/software, covariates, predictor selection, sensitivity analysis, and performance metrics, as well as availability of outputs/code, challenges, and applications. All relevant results in these domains were collected. |
|  | 10b | List and define all other variables for which data were sought (e.g. participant and intervention characteristics, funding sources). Describe any assumptions made about any missing or unclear information. | Methods (p.11-12): Other variables – disease, study region, data source, model type, software, environmental covariates, predictor selection, performance metrics, etc. |
| Study risk of bias assessment | 11 | Specify the methods used to assess risk of bias in the included studies, including details of the tool(s) used, how many reviewers assessed each study and whether they worked independently, and if applicable, details of automation tools used in the process. | Method (p.12): Risk of bias was evaluated by a single reviewer using a structured framework from JBI principles. Each study was assessed across six domains: selection bias, measurement bias, confounding/predictors, analysis/validation, reporting, and overall risk of bias. No automation tools were employed. |
| Effect measures | 12 | Specify for each outcome the effect measure(s) (e.g. risk ratio, mean difference) used in the synthesis or presentation of results. | Not applicable: No quantitative effect measures; narrative synthesis only. |
| Synthesis methods | 13a | Describe the processes used to decide which studies were eligible for each synthesis (e.g. tabulating the study intervention characteristics and comparing against the planned groups for each synthesis (item #5)). | Methods (p.10): Studies grouped by eligibility criteria. |
|  | 13b | Describe any methods required to prepare the data for presentation or synthesis, such as handling of missing summary statistics, or data conversions. | Methods (p 12): Data were standardised (variables, units, covariates) and missing information was recorded as “not reported,” with no imputation. Results were synthesised narratively, tabulated, and visualised in R. |
|  | 13c | Describe any methods used to tabulate or visually display results of individual studies and syntheses. | Results (pp.13–25, Table 1-3, Figures 1–6): Results tabulated and visualised. |
|  | 13d | Describe any methods used to synthesize results and provide a rationale for the choice(s). If meta-analysis was performed, describe the model(s), method(s) to identify the presence and extent of statistical heterogeneity, and software package(s) used. | Methods/Results (pp.12–24): Narrative synthesis; no meta-analysis due to heterogeneity. |
|  | 13e | Describe any methods used to explore possible causes of heterogeneity among study results (e.g. subgroup analysis, meta-regression). | Not performed: Heterogeneity not statistically assessed. |
|  | 13f | Describe any sensitivity analyses conducted to assess robustness of the synthesized results. | No sensitivity analyses were conducted to assess robustness of the synthesized results |
| Reporting bias assessment | 14 | Describe any methods used to assess risk of bias due to missing results in a synthesis (arising from reporting biases). | No statistical or methodological approaches were used to assess risk of bias due to missing results in the synthesis |
| Certainty assessment | 15 | Describe any methods used to assess certainty (or confidence) in the body of evidence for an outcome. | Certainty/confidence not formally assessed; study-level RoB appraised with adapted GRADE/JBI framework. |
| **RESULTS** | | |  |
| Study selection | 16a | Describe the results of the search and selection process, from the number of records identified in the search to the number of studies included in the review, ideally using a flow diagram. | Results (p.13, Figure 1): flow diagram with numbers at each stage. |
|  | 16b | Cite studies that might appear to meet the inclusion criteria, but which were excluded, and explain why they were excluded. | Results (p.13): 58 studies were excluded for the following reasons:  non-spatial prediction  not human cases  prediction not at final scale  not primary research  Other (duplicates/erratum) |
| Study characteristics | 17 | Cite each included study and present its characteristics. | Results (pp.12–24, Table 3, Supplementary S2 File): Study characteristics described. |
| Risk of bias in studies | 18 | Present assessments of risk of bias for each included study. | Results (p.25, Supplementary file 3), Each study’s risk of bias was assessed using a JBI-adapted framework across domains, with ratings from low to high and reviewer notes documenting limitations. |
| Results of individual studies | 19 | For all outcomes, present, for each study: (a) summary statistics for each group (where appropriate) and (b) an effect estimates and its precision (e.g. confidence/credible interval), ideally using structured tables or plots. | Results (pp.12–24, Supplementary 2file : Summarised by disease, method, covariates, data source. |
| Results of syntheses | 20a | For each synthesis, briefly summarise the characteristics and risk of bias among contributing studies. | Results (pp.12–24): Most studies used spatial models with passive or survey data; risk of bias was generally moderate due to limited validation and missing behavioral predictors. |
|  | 20b | Present results of all statistical syntheses conducted. If meta-analysis was done, present for each the summary estimate and its precision (e.g. confidence/credible interval) and measures of statistical heterogeneity. If comparing groups, describe the direction of the effect. | Not applicable: No meta-analysis conducted. |
|  | 20c | Present results of all investigations of possible causes of heterogeneity among study results. | Not applicable: No heterogeneity analysis conducted. |
|  | 20d | Present results of all sensitivity analyses conducted to assess the robustness of the synthesized results. | No sensitivity analyses were conducted to assess the robustness of the synthesized results. |
| Reporting biases | 21 | Present assessments of risk of bias due to missing results (arising from reporting biases) for each synthesis assessed. | Not reported: No reporting bias assessment conducted. |
| Certainty of evidence | 22 | Present assessments of certainty (or confidence) in the body of evidence for each outcome assessed. | Not reported: No certainty/confidence assessment conducted. |
| **DISCUSSION** | | |  |
| Discussion | 23a | Provide a general interpretation of the results in the context of other evidence. | Discussion (pp.25–28): Results interpreted in relation to WHO frameworks and existing knowledge. |
|  | 23b | Discuss any limitations of the evidence included in the review. | Discussion (p.27): Evidence limitations – surveillance data gaps, underreporting, pseudoabsence reliance. |
|  | 23c | Discuss any limitations of the review processes used. | Discussion (p.25): Review limitations – exclusion of grey literature, potential publication bias. |
|  | 23d | Discuss implications of the results for practice, policy, and future research. | Discussion (pp.25): Implications for practice, policy, and research discussed. |
| **OTHER INFORMATION** | | |  |
| Registration and protocol | 24a | Provide registration information for the review, including register name and registration number, or state that the review was not registered. | Registration and Protocol (p27)  The protocol for this systematic review was registered with the Open Science Framework (OSF) **Registration DOI**  <https://doi.org/10.17605/OSF.IO/H6B5N> |
|  | 24b | Indicate where the review protocol can be accessed, or state that a protocol was not prepared. | The protocol for this systematic review was registered with the **Open Science Framework (OSF)** (registration DOI: [10.17605/OSF.IO/H6B5N](https://doi.org/10.17605/OSF.IO/H6B5N)). The full protocol is publicly accessible at the registration link. |
|  | 24c | Describe and explain any amendments to information provided at registration or in the protocol. | Amendments were made to the registered protocol to expand inclusion criteria to all relevant modeling approaches (logistic regression, machine learning, and Bayesian methods) and to include spatial risk mapping as an additional outcome. Additional databases (Ovid) were added to enhance comprehensiveness. |
| Support | 25 | Describe sources of financial or non-financial support for the review, and the role of the funders or sponsors in the review. | Acknowledgments (p.30): Funded by NIHR Global Health Research Unit Programme. |
| Competing interests | 26 | Declare any competing interests of review authors. | Not explicitly declared. |
| Availability of data, code and other materials | 27 | Report which of the following are publicly available and where they can be found: template data collection forms; data extracted from included studies; data used for all analyses; analytic code; any other materials used in the review. | Discussion (p.27): Mention of R and Excel; some studies shared code, but authors did not share their own data/code. |

*From:*  Page MJ, McKenzie JE, Bossuyt PM, Boutron I, Hoffmann TC, Mulrow CD, et al. The PRISMA 2020 statement: an updated guideline for reporting systematic reviews. BMJ 2021;372:n71. doi: 10.1136/bmj.n71. This work is licensed under CC BY 4.0. To view a copy of this license, visit <https://creativecommons.org/licenses/by/4.0/>
