# Supplementary material for: Modelling approaches for predicting the distribution of skin NTDs: A systematic review
Source: PLoS Negl Trop Dis. 2026 May 4;20(5):e0013662. doi: 10.1371/journal.pntd.0013662 (PMC13155686; doi:10.1371/journal.pntd.0013662)
Supplement: Attachment — Submitted filename: Response to Reviewers.docx [file pntd.0013662.s006.docx]

# UNIVERSITY OF KHARTOUM

## FACULTY OF Geographical and Environmental Sciences

## p.o. Box 321Khartoum

## Postal Code: 11115

Mesoud A. Bushara Malik

Lecturer. at Dept. of GIS & Remote Sensing

Faculty of Geographical and Environmental Sciences

University of Khartoum

[mesoudozail@gmail.com](mailto:mesoudozail@gmail.com)

[mesoud.bushara@uofk.edu](mailto:mesoud.bushara@uofk.edu)

+249960666789

April 9, 2026

**Dr**. Shaden Kamhawi, co-Editor-in-Chief, PLOS Neglected Tropical Diseases

**Dr**. Paul Brindley, co-Editor-in-Chief, PLOS Neglected Tropical Diseases

**Re: Author response to reviewers’ comments on manuscript: “Modelling Approaches for Predicting the Distribution of Skin NTDs: A Systematic Review” (Manuscript ID: PNTD-D-25-01471)**

Dear Dr. Kamhawi,

Thank you for considering our manuscript for publication in PLOS Neglected Tropical Diseases. We are grateful to the reviewers for their time, expertise, and constructive feedback. We believe that the revisions we have made in response to their comments have strengthened the manuscript considerably. We have uploaded both a clean version and a tracked‑changes version of the revised manuscript to the Editorial Manager system.

We hope that the revised manuscript now meets the standards for publication in your journal.

Regards,

Mesoud A. Bushara

**Please find below our point‑by‑point responses to the comments.**

We sincerely thank the Editor and Reviewers for their careful review of our manuscript and for the insightful and constructive comments provided. We have carefully considered each comment and revised the manuscript accordingly. We believe that these revisions have strengthened the quality and clarity of the manuscript, and we greatly appreciate your valuable contributions.

**Response to Reviewers**

**Reviewer #1:**

**Comment**: This is a very well written article. I really have only minor comments which are mostly optional.
**Response:** We thank the reviewer for their positive summary and clear suggestions.

**Comment:1.** Line 82- I think I would scabies have been reported to be seasonal in some settings but not others (for example there doesn’t appear to be any meaningful seasonal influence in the Pacific).

**Response**: Thank you for this observation. We have revised the text to clarify that scabies show seasonal variation in certain regions (e.g., Africa) but not in others (e.g., Pacific). The updated sentence now reads: “*scabies has been reported to be seasonal in some settings, while in others no meaningful seasonal influence has been observed*”

**Comment:2.** Geostatistical models were used in 34 and ecological in 33. What was used in the other 2 models?

Response: We thank the reviewer for this observation. The discrepancy was due to a counting error in our initial draft. After correction, the dataset includes 35 geostatistical models and 33 ecological niche models. No additional modelling category was used, and one study that was a preprint has been removed. The Results section has been updated accordingly to state: “*35 geostatistical models and 33 ecological niche models.*”

**Comment:**3. Did you look to see where the modelling work was being done? i.e. are these people from the global north doing all the modelling?

**Response:** We thank the reviewer for raising this important point. To address it, we examined the institutional affiliations of lead authors across all included modelling studies. Our analysis shows that lead authorship is almost evenly split between institutions in the Global North (36 studies) and the Global South (32 studies). This finding challenges the assumption that modelling work is exclusively driven by Northern institutions. Importantly, many of these studies reflect active collaborations between researchers in the Global North and South, highlighting the value of shared expertise. Ethiopia, for example, stands out with seven lead author contributions, underscoring not only the growing modelling capacity within African research institutions but also the role of international partnerships in strengthening local expertise and advancing global health research. We have added a figure (Fig. 4) showing the geographic distribution of lead author institutions by country. This visualization highlights both the dominance of countries such as the UK and USA, and the significant contributions from countries in Africa, Asia, and Latin America. In addition, we have incorporated a description in the Discussion section to highlight the implications of these findings for capacity building, emphasizing the importance of equitable collaborations and sustained investment in endemic regions.

**Reviewer #2:**

This review examines how predictive modelling has been applied to map the distribution of skin NTDs, analyses the types of approaches used and their suitability for informing public health surveillance and control strategies. It also analyses existing evidence gaps and opportunities to strengthen their contribution to planning and decision-making at the program level. The methodology used is sound both for the study design and its registration, following good practices for systematic reviews. Description of results and discussion is easy to follow and presented in a manner that can contribute to improving the application of modelling. I would like to share just a few minor comments aimed at improving the accessibility of this work for readers who may be less familiar with modelling approaches, and for its clarity

**Comment**: 1. A table describing the main characteristics/differences of the ecological models ENM, SDM and risk mapping would be useful.

**Response:**
We thank the reviewer for this constructive suggestion. We agree that it is useful for readers to know how these terms are applied. Given the variation in the use of these terms, we feel the nuances are better captured in text, and have added further explanation to the paragraph starting line 105 on page 7

**Comment**:2. Explain why the search was restricted to year 2000 onwards.

**Response**: We appreciate the reviewer’s query. The restriction to studies published from 2000 onwards was intentional, as this period marks the expansion of spatial epidemiology and the widespread availability of geospatial tools, digital disease data, and environmental datasets (e.g., satellite imagery, climate layers). Earlier studies rarely employed modern predictive modelling approaches or accessible geospatial platforms, making them less comparable to contemporary methods. Limiting the search to 2000 and onwards ensured methodological consistency and relevance to current public health applications. We have added this explanation to the Methods section “*We restricted the search to studies published from 2000 onwards, as this period marks the expansion of spatial epidemiology and the widespread availability of geospatial tools, digital disease data, and environmental datasets. Earlier studies rarely employed modern predictive modelling approaches or accessible geospatial platforms, making them less comparable to contemporary methods. This restriction ensured methodological consistency and relevance to current public health applications”*

**Comment**: 3. Languages included – explain why English, French, Portuguese, Spanish, and Turkish.

**Response:** We thank the reviewer for this observation. These languages were selected based on the expertise of the review team and their relevance to regions where skin NTDs are prevalent. English, French, Portuguese, and Spanish cover the majority of scientific output from Africa, Latin America, and Asia, while Turkish was included given contributions from Turkey and neighboring regions. This ensured broad coverage of the literature while keeping the review feasible. This justification has been added to the Methods section.”*Studies in English, French, Portuguese, Spanish, and Turkish were included, chosen for their relevance to endemic regions and the expertise of the review team.* “

**Editorial Requirements:**

We have completed all requested technical updates:

- Credits author contributions updated for all authors.
- Supporting Information legends added.
- Figure 3 basemap replaced with Natural Earth shapefile, with license terms cited in the legend.
- Financial Disclosure revised to include initials, funding roles, and funder involvement.
- Data Availability tables added (included/excluded studies, extraction details, risk of bias assessments).
